# Peer review of "A Polling-Based Transmission Scheme Using a Network Traffic Uniformity Metric for Industrial IoT Applications"

_sensors, 2019, doi:10.3390/s19010187_

Reviewer 1 Report

The paper deals with the use of Genetic Algorithm for computing communication opportunities in polling-based protocols. In particular, the aim is to uniformly assign communication resources to both periodic and aperiodic traffic.

In the following my observation about the paper:

-          The novelty and advantages of the proposed approach should be better detailed; GA is a well-known technique widely used for solving scheduling problems; the reference section must be improved accordingly.

-          What are advantages of the proposed solution with respect to other approaches, e.g. as simulated annealing?

-          Provide some additional comments about Figure 3; does it refer to the centralized controller?

-          Section 4 is not so easy to read; I suggest to insert a table resuming all the terms and provide a clear description of each one; the proposed examples, shown in the figures 4-5 should be better detailed; what is taug in (2)?what is Ti? What is the node identification number? (a unique id?)

-          Figure 6 is never referenced.

-          How is computed the “pass quality” introduced in Section 6?

-          The simulation engine must be better detailed; how is the routing protocol implemented? Do the authors consider a multi-hop network?

-          The PER is fixed, but table 1 and 2 reports a range 0-20%; what is the actual value? How many simulation runs do the authors consider?

-          What about the effect of initial random selection? Do the authors verify the impact on the GA algorithm?

-          What is the fitness value in Section 7.2?

-          What is the slot information plotted in Figure 11?

-          How is the success probability computed (is it the pass quality as well?)?

-          How do the authors choose the 3s duration of each slot?

-          Why the data collection cycles scale linearly with the number of nodes in the IWSN?

Please, use “s” instead of “sec” (according to the SI standard unit of measurement).

Author Response

We thank the referee for fruitful comments and suggestions. We were pleased to know that our manuscript was rated as potentially acceptable for publication in the special issue, subject to adequate revision and response to the comments raised by reviewer 1.  

As you notice, we have revised the manuscript by modifying the almost all sections. Accordingly, we have uploaded a copy of the original manuscript marked with main changes made during the revision process.

Our responses to the referee's comments are as follows:

--------------------------------------------------------------------------------------------------

(1)The novelty and advantages of the proposed approach should be better detailed; GA is a well-known technique widely used for solving scheduling problems; the reference section must be improved accordingly.

--------------------------------------------------------------------------------------------------

We revised our contoribution of this paper in Introduction and Section 3(Related work). Also, we add description about GA in Section 3.

We think our main contribution of this paper is to propose a data traffic control scheme for polling based communications and verify its performance from viewpoints of end-to-end communication success probability and balanced slot utilization. We adopt a GA as a heuristic to derive an optimal schedule. GA is one of probabilistic search algorithms and optimization techniques based on the mechanisms of natural selection and evolution.

--------------------------------------------------------------------------------------------------

(2)What are advantages of the proposed solution with respect to other approaches, e.g. as simulated annealing?

--------------------------------------------------------------------------------------------------

Although other heuristic algorithms such as simulated annealing, PSO (particle swarm optimization), or even machine learning can also be adopted, we consider a GA-based algorithm in this paper, which could achieve a reasonable and feasible schedule with practically short computation time on an off-the-self computer.

We added this in Section 3. In addition, we chenged the title of Section 6:

  1st draft: GA based data collection algorithm

  Revised draft: GA-based slot assignment algorithm

--------------------------------------------------------------------------------------------------

(3)Provide some additional comments about Figure 3; does it refer to the centralized controller?

--------------------------------------------------------------------------------------------------

We revised Figure 3 and its caption, and added a sentence “A center node has the data traffic control function in order to manage all polling traffic.” 

--------------------------------------------------------------------------------------------------

(4)Section 4 is not so easy to read; I suggest to insert a table resuming all the terms and provide a clear description of each one; the proposed examples, shown in the figures 4-5 should be better detailed; what is taug in (2)?what is Ti? What is the node identification number? (a unique id?)

--------------------------------------------------------------------------------------------------

In Section4, we provided terminologies at first. We inserted a new table (Table 1) to summarize terminlogies and notations. Then, we presented an outline of how our data traffic control scheme for periodic data collection works using Figure 4 and Figure 5.

--------------------------------------------------------------------------------------------------

(5)Figure 6 is never referenced.

--------------------------------------------------------------------------------------------------

At first, we added the label such as “Section 6.1” of each process in Figure 6. Then, we reffereed Figure 6 when we describe the specific process in Section 6.

--------------------------------------------------------------------------------------------------

(6)How is computed the “pass quality” introduced in Section 6?

--------------------------------------------------------------------------------------------------

In Section 6.1.2, we newly added the following sentenses;

“The number of retries depends on a path quality (end-to-end PER) between a node and a central node. A central node gets the path quality from a network manager in IWSN, because a network manager perceives network conditions.”

We think that our scheduler can get network infomration(condition) from a network manager of IWSN because IWSNs such as ISA100.11a or WirelessHART is controled by a centralized device(network manager).

--------------------------------------------------------------------------------------------------

(7)The simulation engine must be better detailed; how is the routing protocol implemented? Do the authors consider a multi-hop network?

--------------------------------------------------------------------------------------------------

We descibed our simlation engine in Section 7.1.(Simulation settings) and also explained how to create route from nodes to a central node. We assume that our scheme can work in standard IWSNs. Standard IWSN such as ISA100.11a support multi-hop network. Therefore, we also consider a multi-hop network.

--------------------------------------------------------------------------------------------------

(8)The PER is fixed, but table 1 and 2 reports a range 0-20%; what is the actual value? How many simulation runs do the authors consider?

--------------------------------------------------------------------------------------------------

In our simulations, we determined PER of a path quality at random for every node. As shown in Table 3, we set the range of PER to 0 − 20%(e.g., PER of node 1 is 2% and PER of node 2 is 14%). Although the link PER dynamically changes in reality, we assume it is stable and constant in this paper.

--------------------------------------------------------------------------------------------------

(9)What about the effect of initial random selection? Do the authors verify the impact on the GA algorithm?

--------------------------------------------------------------------------------------------------

In Section 6.1., we added the following sentences;

We think that the random selection provides a search diversity. Our simulation experiment showed that it did not have a big impact on the performance.”

--------------------------------------------------------------------------------------------------

(10)What is the fitness value in Section 7.2?

--------------------------------------------------------------------------------------------------

The fitnee value is calculated by Equation(13). We have already decribed our fitness function and fitness vlues in Section 5 and in Section 6.

Since it is easy for readers to understand the value, we descrived what is the fitness and how it is calcurated, in Section 7.2 again.

--------------------------------------------------------------------------------------------------

(11)What is the slot information plotted in Figure 11?

-------------------------------------------------------------------------------------------------

We are sorry for the mistake in previous manuscript. That was erroneous description. We revised labels of y-axis of Figure 11, 14, 16.:

 Before: slot information

 After : slot type

--------------------------------------------------------------------------------------------------

(12)How is the success probability computed (is it the pass quality as well?)?

--------------------------------------------------------------------------------------------------

We calculate success probability by Equation(1). Therfore, we added the following sentense in Section 7.1.. 

“The E2E communication success provability is calculated by the left-side hand of Equation (1). “

In addition, we added that path quality is as well as End-to-end PER in Section 6.1.2(Initializaion).

--------------------------------------------------------------------------------------------------

(13)How do the authors choose the 3s duration of each slot?

-------------------------------------------------------------------------------------------------

In Section 4, we explained how we decide the value as follows;

“We consider bandwidth of downward traffic to define polling cycle ∆ts. For example, an our preliminary experiment showed downward traffic for SmartMesh IP that is based on the 6LoWPAN and IEEE802.15.4e standards is assigned every about 2 seconds by the default setting. ∆ts should be longer than the frequency for pre-assigned downlink traffic of IWSN protocols. In this paper, we set ∆ts to 3s for simulation setting.”

--------------------------------------------------------------------------------------------------

(14)Why the data collection cycles scale linearly with the number of nodes in the IWSN?

--------------------------------------------------------------------------------------------------

Our proposal should determin the length of QF that depenend on the number of nodes in an IWSN. Therfore, we descirbed it in Section8.2. as follows;

“At the same time, we expand polling cycles because our proposal data traffic control scheme is like a TDMA manner. To be more precise, we enlarged the length of a QF 10 times because the length of a QF is ∆TQF(= ∆ts × Nall) as described in Section 4. Accordingly, we also enlarged the length of a SF 10 times. Simulation conditions are described in Table 4.”

--------------------------------------------------------------------------------------------------

(15)Please, use “s” instead of “sec” (according to the SI standard unit of measurement).

--------------------------------------------------------------------------------------------------

We corrected the mistake.

We hope the revised version is suitable for publication and look forward to hearing from you in due course.

Sincerely,

Yuichi Igarashi

Reviewer 2 Report

This paper presents a polling-based transmission scheme using a network traffic uniformity metrics for Industry 4.0 or Industrial Internet of Things applications.

In the following, I list my suggestions. The authors may consider to revise the paper.

-   It is difficult to find how the proposed work is different from the existing approaches. The authors presents related work in Section 3. However, I would advise to write a short note in the introduction section to present – how the proposed work is unique with respect to the existing approaches and standards.

-   I would encourage to compare a related work with the proposal in a table for better clarity (line 185 -193).

-   Line 66 – 74 is too detailed for Introduction section. The contribution should be enumerated in very clear manner.

-   The authors may consider to revise contributions, providing an emphasis on – why the contributions is important? Why it is difficult to address from a research standpoint of view.

-   There is a text formatting issues-  unnecessary paragraphs results into poor readability. For instance, line 195 and 196, the text should be grouped into one single paragraph, instead of presenting them in two different lines and as paragraphs.

-   Section 6.1 needs some text, describing the overall objectives of the section, before it moves to Section 6.1.1.

-   Section 7 needs the description of tools and technologies used to perform experiments. The authors may consider to mention the rationales behind of choosing these tools.

Author Response

We thank the referee for fruitful comments and suggestions. We were pleased to know that our manuscript was rated as potentially acceptable for publication in the special issue, subject to adequate revision and response to the comments raised by reviewer 2.

As you notice, we have revised the manuscript by modifying the almost all sections. Accordingly, we have uploaded a copy of the original manuscript marked with main changes made during the revision process.

Our responses to the referee's comments are as follows:

--------------------------------------------------------------------------------------------------

(1)It is difficult to find how the proposed work is different from the existing approaches. The authors presents related work in Section 3. However, I would advise to write a short note in the introduction section to present – how the proposed work is unique with respect to the existing approaches and standards.

--------------------------------------------------------------------------------------------------

In Introduction and Section 3(Realate work), we revised our contribution. We think our main contribution of this paper is to propose a data traffic control scheme for polling based communications and verify its performance from viewpoints of end-to-end communication success probability and balanced slot utilization. We adopt a GA as a heuristic to derive an optimal schedule. GA is one of probabilistic search algorithms and optimization techniques based on the mechanisms of natural selection and evolution.

--------------------------------------------------------------------------------------------------

(2)I would encourage to compare a related work with the proposal in a table for better clarity (line 185 -193).

--------------------------------------------------------------------------------------------------

We inserted a new table that summarize related work in comparison with our proposal in Section 3(Related work). In addtion, we revised introduction of related work and accordingly clarified and revised our contribution of this paper in Section 3.

--------------------------------------------------------------------------------------------------

(3)Line 66 – 74 is too detailed for Introduction section. The contribution should be enumerated in very clear manner.

--------------------------------------------------------------------------------------------------

We revised the contribution part of Introduction as mentioned above.

--------------------------------------------------------------------------------------------------

(4)The authors may consider to revise contributions, providing an emphasis on – why the contributions is important? Why it is difficult to address from a research standpoint of view.

--------------------------------------------------------------------------------------------------

As mentioned above(1),(2) and (3), we revised our contributrion of this paper.

--------------------------------------------------------------------------------------------------

(5)There is a text formatting issues-  unnecessary paragraphs results into poor readability. For instance, line 195 and 196, the text should be grouped into one single paragraph, instead of presenting them in two different lines and as paragraphs.

--------------------------------------------------------------------------------------------------

We revised the text formatting issue.

--------------------------------------------------------------------------------------------------

(6)Section 6.1 needs some text, describing the overall objectives of the section, before it moves to Section 6.1.1.

--------------------------------------------------------------------------------------------------

We newly added some text in Section 6.1. We described our problems of timeslot assignement at first. Then, we persent what information we encode as chromosomes before moving to Section 6.1.1..

--------------------------------------------------------------------------------------------------

(7)Section 7 needs the description of tools and technologies used to perform experiments. The authors may consider to mention the rationales behind of choosing these tools.

--------------------------------------------------------------------------------------------------

We descibed our simlation engine in Section 7.1.(Simulation settings) and also, we additionaly mentioned we use GA as a heuristic to derive an optimal schedule in Section 3(Related Work).

We hope the revised version is suitable for publication and look forward to hearing from you in due course.

Sincerely,

Yuichi Igarashi

Reviewer 3 Report

In the context of Industrial IoT (IIoT), authors presented a data traffic control scheme over IWSNs for polling-based data collection and its data collection scheduling. Paper is scientifically sound, organization linear, motivations well introduced, research area topical. In particular, the usage of genetic algorithm is well explained and simulations set satisfactory. However, manuscript needs some interventions and a minor review. First of all, a deep proof-reading in order to fix minors (e.g. "all of the" ->"all the", missing space in "PA(Process" and in "phase.We";consider introducing IIoT acronym; "Fig.1 an example"->"Fig.1 An example" and same for Fig.2 caption; [10][11][12][13][14][15]->[10-15]), typos ("number of retransmission" ->"number of retransmissions"; "each areas";"this problem have"; "each individuals"; "provability") and re-phrase some statements for the sake of redability (e.g., " show that both network traffic is generated uniformly and a center node can collect periodic data from nodes at high success ratio which the average probability is 97.4% and the lowest one is 95.2%";"Both application data packets, and network control packets to maintain the wireless network, are transmitted"; use past tense verbs in conclusion "we do not address"). Then, provide more details about software exploited for simulations and scenario setting. Insert predictive maintanace among the conventional IIoT application goals. Edit flowchart of Fig.6 to avoid double "Mutation" label. Provide a comment and data (if available) about the energetic profile of the proposed data traffic control scheme: since it is tailored on IWSN and polling/broadcast are energy demanding practices, such aspect cannot be disregarded (as undelrined in <Phuong, Tran Minh, and Dong Seong Kim. "Efficient power control scheme for cognitive industrial sensor networks." International Journal of Control and Automation 7.3 (2014): 177-188.>). It is a crucial point,in my opinion. Improve bibliography: cite<G. Fortino, C. Savaglio and M. Zhou, "Toward opportunistic services for the industrial Internet of Things," 2017 13th IEEE Conference on Automation Science and Engineering (CASE), Xi'an, 2017, pp. 825-830.>to emphasize the impact on IIoT and its numbers at line 19,and <Gubbi, Jayavardhana, et al. "Internet of Things (IoT): A vision, architectural elements, and future directions." Future generation computer systems 29.7 (2013): 1645-1660.> as general references for IWSN at line 39; finally <> as general reference for IoT at line 16. In conclusion, paper has merit but the aforementioned issues need to be addressed for its fully acceptance.

Author Response

We thank the referee for fruitful comments and suggestions. We were pleased to know that our manuscript was rated as potentially acceptable for publication in the special issue, subject to adequate revision and response to the comments raised by the reviewer 3.

As you notice, we have revised the manuscript by modifying the almost all sections. Accordingly, we have uploaded a copy of the original manuscript marked with main changes made during the revision process.

Our responses to the referee's comments are as follows:

-

--------------------------------------------------------------------------------------------------

(1)First of all, a deep proof-reading in order to fix minors 

e.g. "all of the" ->"all the", 

missing space in "PA(Process" and in "phase.We";

consider introducing IIoT acronym; 

"Fig.1 an example"->"Fig.1 An example" and same for Fig.2 caption; 

[10][11][12][13][14][15]->[10-15]), 

typos ("number of retransmission" ->"number of retransmissions";"each areas";"this problem have"; "each individuals"; "provability")

re-phrase some statements for the sake of redability (e.g., " show that both network traffic is generated uniformly and a center node can collect periodic data from nodes at high success ratio which the average probability is 97.4% and the lowest one is 95.2%";"Both application data packets, and network control packets to maintain the wireless network, are transmitted"; 

use past tense verbs in conclusion "we do not address"). 

--------------------------------------------------------------------------------------------------

We corrected grammatical mistakes and some typos.

--------------------------------------------------------------------------------------------------

(2)Then, provide more details about software exploited for simulations and scenario setting.

--------------------------------------------------------------------------------------------------

We descibed our simlation engine in Section 7.1.(Simulation settings) and also explained how to create route from nodes to a central node. We assume that our scheme can work in standard IWSNs. Standard IWSN such as ISA100.11a support multi-hop network. Therefore, we also consider a multi-hop network.

--------------------------------------------------------------------------------------------------

 (3)Insert predictive maintanace among the conventional IIoT application goals.

--------------------------------------------------------------------------------------------------

In Introduction, we inserted “predictive maintanace” as one of Industrial applications to operate systems more efficiently and economically.

--------------------------------------------------------------------------------------------------

 (4)Edit flowchart of Fig.6 to avoid double "Mutation" label. 

--------------------------------------------------------------------------------------------------

At first, we added the label such as “Section 6.1” of each process in Figure 6. Then, we reffereed Figure 6 when we describe the specific process in Section 6.

--------------------------------------------------------------------------------------------------

(5)Provide a comment and data (if available) about the energetic profile of the proposed data traffic control scheme: since it is tailored on IWSN and polling/broadcast are energy demanding practices, such aspect cannot be disregarded (as undelrined in). It is a crucial point,in my opinion. 

--------------------------------------------------------------------------------------------------

We agree with your opinion. We added new section(8.3 Power consumption) to Section 8(Discussion). We think that we have to evalate power consumption details but it is one of our future work. We specified our opinion and our future problem in Section 8.3.

--------------------------------------------------------------------------------------------------

(6)Improve bibliography: 

cite<G. Fortino, C. Savaglio and M. Zhou, "Toward opportunistic services for the industrial Internet of Things," 2017 13th IEEE Conference on Automation Science and Engineering (CASE), Xi'an, 2017, pp. 825-830.>to emphasize the impact on IIoT and its numbers at line 19, 

andas general references for IWSN at line 39;

finally <> as general reference for IoT at line 16.

--------------------------------------------------------------------------------------------------

We cited all papers that you suggested. Also, we cited the following three documents as general reference for IoT;

<N. Gershenfeld, R. Krikorian, and D. Cohen, The Internet of Things. Scientific American2004, 291, 76-81.

ITU Internet Reports, The Internet of Things: 7th Edition. www.itu.int/internetofthings/on>

<G. Fortino, C. Savaglio and M. Zhou, Toward opportunistic services for the industrial Internet of Things,

13th IEEE Conference on Automation Science and Engineering (CASE) 2017, 825-830>

We hope the revised version is suitable for publication and look forward to hearing from you in due course.

Sincerely,

Yuichi Igarashi

Reviewer 4 Report

This article describes a data traffic control scheme over IWSNs for collecting data based on polling, using industrial IoT applications. IoT technologies are used in social infrastructure and industrial applications, like process automation, distribution automation or advanced metering infrastructure, to improve the efficiency in the production process, increase the efficiency and ensure the optimisation of resource consumption. The described algorithms provide a uniform network traffic load for periodical data collections and all nodes reply a response packet corresponding to a polling request. 

This article can raise the interest of people working in industrial IoT and polling based communication protocols, and it is an up-to-date kind of study, where in-depth details of the used methodology are provided. All the specific terms are well-explained, and technical readers can easily understand the overall concepts discussed.

The IoT technologies can help us improve resource consumption and production efficiency. Some protocols have been developed in the recent years that can improve the communication reliability in wireless network. The article propose a data traffic control scheme that can help achieving both heterogeneous periodic data collection with high success rate and unpredictable on-demand communication. The scheme is used for polling based data collection from multiple industrial IoT applications and its data collection scheduling. Three kinds of slot types are used to decide a schedule, one for transmitting a polling query, one for transmitting a retry packet in case of wireless communication failure or an unpredictable on-demand request and one for letting some slots open for unpredictable on-demand requests. 

I think that the authors should focus a bit more on explaining the importance of the research for a practical use case.

The references are diverse but not updated (i.e. 2017, 2018), more details about time critical aspects in IoT should be added, for example:

- Igarashi, Yuichi, Yoshiki Matsuura, Minoru Koizumi, and Naoki Wakamiya. "Priority-based dynamic multichannel transmission scheme for industrial wireless networks." Wireless Communications and Mobile Computing 2017 (2017).

- Zhao, Zhiming, Paul Martin, Andrew Jones, Ian Taylor, Vlado Stankovski, Guadalupe Flores Salado, George Suciu, Alexandre Ulisses, and Cees de Laat. "Developing, Provisioning and Controlling Time Critical Applications in Cloud." In European Conference on Service-Oriented and Cloud Computing, pp. 169-174. Springer, Cham, 2017.

- Haxhibeqiri, Jetmir, Eli De Poorter, Ingrid Moerman, and Jeroen Hoebeke. "A Survey of LoRaWAN for IoT: From Technology to Application." Sensors 18, no. 11 (2018): 3995.

Grammar & spelling check:

- misspelled words: monocycle, consecutive, data link, conflict-free, real-time, polling.

- missing articles: the above difficulties, the real field, a wireless network, etc

Also, punctuation should be revised.

Author Response

We thank the referee for fruitful comments and suggestions. We were pleased to know that our manuscript was rated as potentially acceptable for publication in the special issue, subject to adequate revision and response to the comments raised by the reviewer. 

As you notice, we have revised the manuscript by modifying the almost all sections. Accordingly, we have uploaded a copy of the original manuscript marked with main changes made during the revision process.

Our responses to the referee's comments are as follows:

-

--------------------------------------------------------------------------------------------------

I think that the authors should focus a bit more on explaining the importance of the research for a practical use case.

--------------------------------------------------------------------------------------------------

In Introduction and Section 3(Realate work), we revised our contribution. We think our main contribution of this paper is to propose a data traffic control scheme for polling based communications and verify its performance from viewpoints of end-to-end communication success probability and balanced slot utilization. We adopt a GA as a heuristic to derive an optimal schedule. GA is one of probabilistic search algorithms and optimization techniques based on the mechanisms of natural selection and evolution.

--------------------------------------------------------------------------------------------------

The references are diverse but not updated (i.e. 2017, 2018), more details about time critical aspects in IoT should be added, for example:

- Igarashi, Yuichi, Yoshiki Matsuura, Minoru Koizumi, and Naoki Wakamiya. "Priority-based dynamic multichannel transmission scheme for industrial wireless networks." Wireless Communications and Mobile Computing 2017 (2017).

- Zhao, Zhiming, Paul Martin, Andrew Jones, Ian Taylor, Vlado Stankovski, Guadalupe Flores Salado, George Suciu, Alexandre Ulisses, and Cees de Laat. "Developing, Provisioning and Controlling Time Critical Applications in Cloud." In European Conference on Service-Oriented and Cloud Computing, pp. 169-174. Springer, Cham, 2017.

- Haxhibeqiri, Jetmir, Eli De Poorter, Ingrid Moerman, and Jeroen Hoebeke. "A Survey of LoRaWAN for IoT: From Technology to Application." Sensors 18, no. 11 (2018): 3995.

--------------------------------------------------------------------------------------------------

We cited all papers that you suggested.

--------------------------------------------------------------------------------------------------

Grammar & spelling check:

- misspelled words: monocycle, consecutive, data link, conflict-free, real-time, polling.

- missing articles: the above difficulties, the real field, a wireless network, etc

Also, punctuation should be revised.

--------------------------------------------------------------------------------------------------

We corrected the grammatical mistakes and some typos.

We hope the revised version is suitable for publication and look forward to hearing from you in due course.

Sincerely,

Yuichi Igarashi

Round  2

Reviewer 1 Report

The authors fulfilled all my previous requests and in my opinion the paper can be accepted in the present form.

Author Response

Dear the reviewer 1,

−−−−−−−−−−−−−−−−−−−−−−−−−−−−−−−−−−−−−−−−−−−−−−−−−−−−−−−−−−−−−−−−−−−−−−−−

English language and style

( ) Extensive editing of English language and style required  
( ) Moderate English changes required  
(x) English language and style are fine/minor spell check required  
( ) I don't feel qualified to judge about the English language and style 

Comments and Suggestions for Authors

The authors fulfilled all my previous requests and in my opinion the paper can be accepted in the present form.

−−−−−−−−−−−−−−−−−−−−−−−−−−−−−−−−−−−−−−−−−−−−−−−−−−−−−−−−−−−−−−−−−−−−−−−−

We corrected grammatical mistakes and some typos. All changes are highlighted in the revised manuscript.

We hope the revised version is suitable for publication.